# The Relationship between Nasal Carriage of *Staphylococcus aureus* and Surgical Site Infections in a Hospital Center in Morocco

**DOI:** 10.1155/2021/5585588

**Published:** 2021-08-26

**Authors:** Rachid Flouchi, Abderrahim Elmniai, Abdelaziz Hibatallah, Karim Fahsi, Ibrahim Touzani, Kawtar Fikri-Benbrahim

**Affiliations:** ^**1**^ Laboratory of Microbial Biotechnology and Bioactive Molecules, Science and Technologies Faculty, Sidi Mohamed Ben Abdellah University, Fez, Morocco; ^2^High Institute of Nursing Professions and Health Techniques Annex Taza, Fez, Morocco; ^3^Human Pathology, Biomedicine and Environment Laboratory, Faculty of Medicine and Pharmacy, Sidi Mohamed Ben Abdellah University, Fez, Morocco; ^4^Provincial Laboratory of Epidemiology and Environmental Hygiene, DMS, Taza, Morocco; ^5^Surgical Department, Provincial Hospital Center Ibn Baja, Taza, Morocco; ^6^High Institute of Nursing Professions and Health Techniques, Rabat, Morocco

## Abstract

**Background:**

Surgical site infection is a major public health problem in the world. Nasal carriage is a major risk factor for the development of nosocomial *Staphylococcus aureus* infection, especially methicillin-resistant *Staphylococcus aureus* (MRSA). Our work aims to determine the prevalence of *Staphylococcus aureus*, methicillin-resistant *Staphylococcus aureus*, and the associated risk factors and to evaluate their sensitivity to 27 antibiotics.

**Methods:**

A cross-sectional study was carried out on 100 patients, hospitalized in preoperative care of surgery units at the Taza Provincial Hospital Center in the Fez-Meknes region, from January to June 2019. Samples were taken from the patient's anterior nostril using single-use sterile dry or wet cotton swabs and then analyzed in the Provincial Public Health Laboratory in Taza. The carriage of *Staphylococcus aureus* was studied by conventional bacteriological methods by spreading nasal swabs on Chapman culture medium, while antibiotic resistance was determined by the Mueller–Hinton agar disc diffusion method according to the recommendations described by the Antibiogram Committee of the French Society of Microbiology 2019 (CA SFM 2019).

**Results:**

Of the 84 patients found to be positive, 45.24% had coagulase-positive *Staphylococcus aureus* and 54.76% had coagulase-negative *Staphylococcus.* After surgery in the postoperative phase, 16 patients developed surgical site infections, of which two had a negative nasal culture and 14 had positive nasal culture. Among the *Staphylococcus aureus*-positive patients, 36.84% were colonized by a methicillin-resistant *Staphylococcus aureus* (MRSA) and 63.16% by a methicillin-sensitive *Staphylococcus aureus* (MSSA). Of these, 57.14% of MRSA colonized patients developed an infection of the surgical site and 42.85% showed no sign of SSI, while for patients colonized by MSSA, 16.67% developed SSI and 83.33% showed no sign of SSI. Moreover, children were the most affected by MRSA. Concerning antibiotic sensitivity, multiresistance of MRSA to more than 3 antibiotics has been found.

**Conclusion:**

To the best of our knowledge, this is the first study carried out in this hospital center with the aim of knowing the prevalence of nasal carriage of *Staphylococcus aureus* and MRSA and to identify the risk factors in order to prevent infections related to nasal carriage of *Staphylococcus aureus* and MRSA.

## 1. Introduction

Surgical site infections (SSIs) are infections that occur after surgery, 30 days postoperatively or one year postoperatively for prosthetic surgery procedures. These infections are classified as superficial, deep, organ, or space infections [1]. SSIs represent a concern for health systems because of their high mortality and morbidity rates, the increase in the average length of hospital stay, as well as expenses and costs, which increases the number of postoperative patients [2, 3] and reduces the care quality and the hospital's branding. SSIs are the second nosocomial infection occuring in France after urinary tract infections [3] and pose a major public health problem in Africa due to their incidence varying between 6.8% and 26% [4]. *Staphylococcus aureus* remains the most common cause of infections in operating sites [5].

In humans, the anterior nostrils of the nose are the most common sites of carriage for *Staphylococcus aureus* (*S. aureus*), which remains a well-defined risk factor for infection with this bacterium [6, 7]. In a previous study on the role of nasal carriage in *S. aureus* infections, it has been shown that there is an increase in carriage rates in extranasal sites for nasal *S. aureus* carriers, for example, skin carriage on the hands increases from 27% in normal population to 90% in permanent nasal *S. aureus* carriers [6], which present in surgery cases a higher risk of nosocomial *S. aureus* infection (bacteremia) compared to controls [8].

Nasal carriage of *S. aureus* creates a major risk of SSI in carrier's patients, some of whom have a greater risk by carrying methicillin-resistant *Staphylococcus aureus* (MRSA), which is an additional risk factor for surgical site infection and has become endemic in some hospital areas [9]. In addition, the ineffectiveness of antibiotic prophylaxis on MRSA strains increases the risk of SSI with MRSA in patients treated in surgical intensive care units [10] in Morocco, where little research has been done on this subject. To this end, the objective of our investigation is to study the nasal carriage of *Staphylococcus aureus* and non-*aureus* in preoperative patients in a provincial hospital center and to study the antibiotic resistance of all strains isolated in order to determine their relationship with postoperative patients' surgical site infection.

## 2. Materials and Methods

### 2.1. Study Population

This is a cross-sectional prospective study which was conducted over a period of six months (January to June 2019) in a Provincial Hospital Center in Taza city (in Fez-Meknes region, in the northeast of Morocco). The study concerned patients hospitalized in preoperative care in the men's, women's, children's, and gynecological-obstetric surgery units. Concerning the inclusion criteria, patients hospitalized preoperatively, having benefited from a surgical intervention and being followed up postoperatively, were included in this study, while the patients who did not benefit from a surgical intervention were all excluded. The choice was made in an anarchic way according to patients who agreed to participate in the study.

Patients' medical records were reviewed and examined for demographic information: for information on the antibiotics prescription in the postoperative period and for the detection of surgical site infections.

### 2.2. Sampling Mode

Samples were taken using single-use sterile dry or moist cotton swabs which were inserted into the patient's anterior nostril (1-2 cm). Thereby, nasal secretions were collected by performing 5 complete rotations of the swab, the same swab being used for both nostrils [11]. Immediately after taking the samples, the swabs were sent directly in an isothermal box to the Provincial Public Health Laboratory in Taza, where they were spread on the Chapman medium and then incubated at 35°C (±2) for 24 to 48 hours.

### 2.3. Bacteriological Analysis

The carrying of *S. aureus* was investigated by classical bacteriological methods by spreading nasal swabs on the Chapman culture medium (OXOÏD society). Colonies fermenting mannitol were suspected as *Staphylococcus aureus*; then, bacterial identification was based on morphological and biochemical characters: colonies appearance; mannitol's fermentation based on the strain's ability to use or not mannitol as carbon source; and DNAse positive, Gram positive, oxidase positive, and catalase negative activities. Hence, creamy colonies having a circular outline, a rounded shape, a smooth appearance, a convex elevation, and a viscous consistency are characteristic to *S. aureus* for which microscopic examination shows Gram-positive diplococci or cocci in regular clusters. Moreover, fermentation produces organic acids after sugar's degradation; the accumulation of these organic acids in the culture medium induces its acidification leading to a change of the phenol red (pH indicator) to yellow. Furthermore, DNase tests are conducted on a DNA agar medium, after 18 to 24 hours of incubation, the presence of a clear area around the streaks indicates the presence of DNase activity.

Finally, the strain's confirmation is carried out on an API^®^ 20 Staph gallery (bioMérieux company) made up of 20 microtubes containing dehydrated substrates. The microtubes are inoculated with a bacterial suspension prepared on the API Staph medium. The reactions produced during the incubation period result in spontaneous color changes or are revealed by the addition of specific reagents; then, identification is made using the analytical catalog. The identified strains were stored at −18°C.

### 2.4. Sensitivity to Antibiotics

Antibiotic resistance was determined by the Mueller–Hinton agar disc diffusion method according to the recommendations described by the Antibiogram Committee of the French Society of Microbiology 2019 (CA SFM 2019) [12]. The Mueller–Hinton medium was inoculated by swabbing pure bacterial strains of *Staphylococcus aureus* and incubated 18–24 hours at 37°C, after placing antibiotic discs (BIOANALYSEⓇ society). The antibiotic discs used with their respective load are as follows: amikacin (30 *μ*g); amoxicillin (25 *μ*g); amoxicillin + clavulanic acid (10 *μ*g); ceftazidime (30 *μ*g); cefixime (10 *μ*g); ceftriaxone (30 *μ*g); chloramphenicol (30 *μ*g); ciprofloxacin (5 *μ*g); cefalexin (30 *μ*g); cefoxitin (30 *μ*g); cefotetan (30 *μ*g); cefotaxime (30 *μ*g); cefalotin (30 *μ*g); erythromycin (15 *μ*g); fusidic acid (10 *μ*g); imipenem (10 *μ*g); lincomycin (15 *μ*g); nalidixic acid (30 *μ*g); ofloxacin (5 *μ*g); oxacillin (5 *μ*g); piperacillin (30 *μ*g); rifampicin (30 *μ*g); trimethoprim + sulfamethoxazole (1.25 *μ*g); tetracycline (30 *μ*g); ticarcillin (75 *μ*g); tobramycin (30 *μ*g); and teicoplanin (30 *μ*g).

The antibiogram reading was done according to the recommendations of CA SFM 2019 [12], and the inhibition diameters were measured manually using a graduated ruler. Hence, methicillin resistance was determined by the diffusion method of the cefoxitin disc, and an inhibition diameter around this disc of less than 22 mm indicates the suspicion of the presence of a methicillin-resistant *Staphylococcus aureus* strain.

### 2.5. Statistical Analysis

Data were analyzed using SPSS and Microsoft Office Excel 2010. The *p* values for the variables analyzed in each case were calculated by the Fisher's exact test and the chi^2^ test, considering *p* value <0.001 as highly significant, *p* value <0.01 as very significant, and *p* value <0.05 as significant.

## 3. Results

For a total of 100 patients, 25% for each service, the mean age of 39 years (±1 year) and sex ratio of 1.22 have been registered. In the preoperative period, 84 (84%) patients have had positive nasal cultures and 16% (*n* = 16) have had negative nasal cultures. Of the 84 patients revealed positive, 45.24% (*n* = 38) has had a coagulase-positive *Staphylococcus aureus*, and 54.76% (*n* = 46), a coagulase-negative *Staphylococcus* (Table 1).

In the postoperative phase, 16 patients have presented surgical site infections, among which 2 patients have had negative nasal culture and 14 have had positive nasal culture (12 patients with *Staphylococcus aureus* and 2 patients with coagulase-negative *Staphylococcus*).

**Figure 1:**
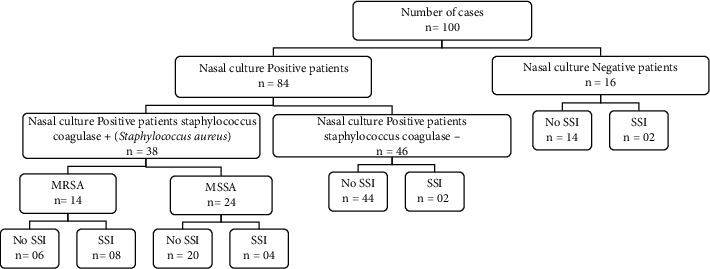
Flowchart of patient distribution according to *Staphylococcus* colonization.

Among the *Staphylococcus aureus*-positive patients, 14 (36.84%) have been colonized with methicillin-resistant *Staphylococcus aureus* (MRSA) and 24 (63.16%) have been colonized with methicillin-sensitive *Staphylococcus aureus* (MSSA). Of these, 8 MRSA colonized patients have developed an operative site infection (SSI) (57.14%) and 6 have shown no signs of SSI (42.85%), and for MSSA colonized patients, 4 (16.67%) have developed an SSI and 20 (83.33%) have shown no signs of SSI (Figure 1).

For the most affected departments (Table 1), the gynecology department have been the most affected with 12 patients colonized by *Staphylococcus aureus* (31.57%), followed by children's surgery (*n* = 11; 28.95%), women's surgery (*n* = 09; 23.69%), and men's surgery (*n* = 06; 15.79%), respectively. The age group most affected by S. *aureus* has been the group between 30 and 45 years old with 13 cases (34.22%), followed by the group under 15 years old with 11 cases (28.95%), and then 8 people over 45 years old (21.04%). Females have been the most carriers of *S. aureus* with a rate of 63.16%. Moreover, patients admitted to the programmed surgery are the most colonized by *S. aureus* with 20 cases (52.63%), as well as patients with no venous catheters and patients not presenting any associated pathology are the most affected by *S*. *aureus*.

Concerning specialties, gynecology has recorded the highest number of *S. aureus* infections, followed by pediatric surgery, orthopedic surgery, visceral surgery, and urology. However, these infections have been absent in otolaryngology surgery and neurosurgery. Otherwise, patients from rural areas have been the main carriers of *S. aureus* with a percentage of 65.78% compared to urban patients. For services, where operative site infection was developed, the children's surgeries service has ranked first with 5 cases (41.66%) followed by gynecology (3 cases) (25.00%) and then women's and men's surgeries having the same cases number (2 cases in each surgey unit corresponding to 16.67%) (Table 1).

Regarding the regression analysis between *S. aureus* and patient characteristics (Table 1), the rate of *S. aureus* carriers has been found statistically insignificant in relation to services, age, sex, venous catheter, and pathologies associated with the specialty and origin (*p* > 0.05). However, a highly significant relationship has been noted between *S. aureus* carriers and surgical site infections, *p*=0.007 (*p* < 0.01), odds ratio at the 95% confidence interval, OR = 4.2391 [1.3016; 15.3893], as well as a significant relationship between *S. aureus* carriers and the nature of admission to services, *p*=0.01, odds ratio at the 95% confidence interval, OR = 3.0483 [1.181; 8.0862].

In relation to MRSA colonized patients (Table 2), the children's surgery department has the highest number of patients (5 cases (35.71%)), followed by men's surgery (4 cases (28.58%)), then gynecology (3 cases (21.43%)) and women's surgery (2 cases (14.28%)). Regarding the age group, most colonized with MRSA, it is under 15 years old (35.71%), while the carriers of MSSA are between 30 and 45 years old (37.50%). Moreover, females are the most affected by MRSA (64.29%), as well as patients admitted in emergency, venous catheter carriers, and also patients without associated pathologies. For the specialty, children's surgery is at the first level (with 5 cases of MRSA) followed by gynecology and orthopedic surgery (3 cases for each one), then visceral (2 cases), and urology (1 MRSA carrier case). Patients from rural areas are the most colonized by MRSA (78.57%). For the services affected by MRSA whose patients developed an SSI, child surgery (37.5%) is followed by gynecology and men's surgery (25%) and finally women's surgery (12.5%).

Concerning the regression analysis between MRSA and patient characteristics (Table 2), MRSA has been found statistically nonsignificant in relation to services, age, sex, venous catheter, and pathologies associated with the specialty and origin (*p* > 0.05), but a significant relationship has been reported between MRSA carriers and surgical site infections, *p*=0.04 (*p* < 0.05), odds ratio at 95% confidence interval, OR = 4.908 [0.9553; 34.6181], and also between MRSA carriers and the nature of admission to services, *p*=0.014 (*p* < 0.05), odds ratio at 95% confidence interval, OR = 6.2793 [1.1926; 39.9359].

Regarding the antibiotic resistance profile (Table 3), out of the 38 *Staphylococcus aureus* isolated strains, 14 have shown total resistance to cefoxitin (MRSA), cefotaxime, and ticarcillin, while MRSA has shown strong resistance to amoxicillin + clavulanic acid (57.14%), amoxicillin (64.29%), ceftazidime (85.72%), ceftriaxone (78.58%), erythromycin (64.29%), cephalothin (85.72%), nalidixic acid (57.15%), oxacillin (92.86%), piperacillin (78.58%), and teicoplanin (85.73%). Furthermore, MRSA has shown a total sensitivity to amikacin, chloramphenicol, ciprofloxacin, cefalexin, imipenem, lincomycin, ofloxacin, rifampicin, trimethoprim-sulfamethoxazole, and tetracycline and also showed a high sensitivity to cefotetan (85.71%), fusidic acid (78.57%), and tobramycin (85.71%), while MSSA has shown a slight resistance to some antibiotics and a high sensitivity to all antibiotics at a rate of over 70%. So, we can conclude that a high prevalence of multiresistance to antibiotics has been noticed for MRSA compared to MSSA which has a slight resistance to some antibiotics and a high sensitivity to all antibiotics.

## 4. Discussion

Nasal carriage is a risk factor for surgical site infections [13]. In our study, 38% of the patients have shown nasal carriers of *S. aureus* (31.57% of them developed an SSI). Eventhough some studies showed lower percentages which did not reach 30%, this result is similar to the results of investigations conducted in Lebanon (38.4%) [14] and Iraq (38.5%) [15] and higher than results reported in Ethiopia (34.58%) [16], Netherland (26.0%) [17], Japan (25%) [18], Spain (20.6%) [19], Algeria (18.3%) [20], and a study carried out at the Mohammed V Military Training Hospital in Morocco (31%) [9].

Concerning the patient's factors and *S. aureus* nasal porting status, a highly significant relation has been observed between *S. aureus* nasal porting and emergency admission mode (*p*=0.01) and a highly significant relationship between nasal porting and surgical site infection (*p* < 0.01). These results confirm those reported by Lepelletier et al. in 2011. However, no relationship has been observed between *S. aureus* nasal porting and other patient factors.

MRSA is one of the major multiresistant organisms and represents a major public health problem in the world [21]. In our study, the MRSA rate is 14% versus 24% of MSSA, and these results differ from those found elsewhere. In fact, it differs from one country to another according to the geographical area. Hence, a rate of 33.8% was reported in Iran [22], 1.2% in Turkey [23], 45.4% in Cameroon [24], 6.8% in Taiwan [25], 40% in Egypt [26], 53.4% in East Africa [27], and 3.4% in Japan [18]. In our study, MRSA is generally observed in patients who have recently been hospitalized and shows a significant relationship between MRSA nasal porting and the admission's mode (*p* < 0.05), which explains why patients admitted to emergency departments have a high risk of MRSA colonization.

In terms of age groups, children under 15 years have recorded a high number of MRSA, which is similar to other studies [28]. The highest MRSA carrier rate is recorded among female patients, which is contradictory to the results found at another hospital center in Morocco [9], where the MRSA carrier rate was higher in the child surgery department followed by the men surgery department. This proportion varies according to the department type and patients categories as well as according to the care type in the different departments.

For surgical site infections, a significant relationship between MRSA and surgical site infections (*p* < 0.05) has been found. This result is similar to those reported by different studies [18, 29, 30].

Regarding antibiotic susceptibility, MRSA isolates show high resistance to ceftazidime (85.72%), cefixime (78.58%), erythromycin (64.29%), Cefalotin (85.72%), piperacillin (78.58%), and teicoplanin (85.73%) which means multidrug resistance. Other studies in Morocco have shown that resistance to Erythromycin and the antibiotic resistance profile varies with geographical region and changes over time [31]. Our multidrug resistance result is consistent with other studies [32, 33], which is explained by the antibiotics overuse on the one hand and by patients self-medication with antibiotics on the other hand.

Nasal carriage of *S. aureus* is a well-identified factor in patients undergoing surgical procedures in surgical departments; hence, it is necessary to eradicate. In this context, some scientific studies carried out showed firstly the effectiveness of a whole body wash with a 4% chlorhexidine solution for the eradication of the MRSA strains [34] and secondly the effectiveness of the antibiotic agent mupirocin [35] in reducing the surgical site infection [35]. Furthermore, the application of decontamination procedures in preoperative patients can be difficult, especially for emergency surgery, due to the time required for diagnosis (2 days for culture) and 3 days for treatment [30].

Despite some limitations of this study concerning mainly the investigation duration, as it was conducted over a period of six months, and the limited patients' numbers (25 patients for each department), which did not enable to differentiate permanent from intermittent nasal carriage in preoperative patients; it appears that nasal carriage of *S. aureus* significantly increases the rate of nosocomial surgical site infection (SSI) after surgery and is an independent risk factor for postoperative wound infections. Therefore, regular examination is required for patients to reduce the number of spontaneous colonization of *S. aureus* and to minimize the risk of surgical site infection.

## 5. Conclusion

This study focuses on the nasal carriage of *S. aureus* and the importance of screening patients for MRSA. Results showed a significant relationship between MRSA and surgical site infections as well as the admission mode of patients to the hospital. Furthermore, in our study, children have been at greater risk of MRSA than other patients. Regarding antibiotic sensitivity, MRSA isolates have been found to develop multiresistance to antibiotics, which requires early detection of MRSA during patient admissions, decolonization of carrier patients, and control of antibiotic prescription in order to prevent nosocomial infections, especially SSI.

## Figures and Tables

**Table 1:** Demographic and clinical characteristics of patients by nasal carriage status of *Staphylococcus aureus* (*N* = 100).

Demographic and clinical characteristics of patients	Carriers of *Staphylococcus aureus, n* = 38	Noncarriers of *Staphylococcus aureus, n* = 62	OR confidence interval at 95%	*p* value
*Services*				
Women's surgery	9 (23.69%)	16 (25.80%)	—	0.86
Men's surgery	6 (15.79%)	19 (30.65%)		
Gynecology	12 (31.57%)	13 (20.97%)		
Child surgery	11 (28.95%)	14 (22.58%)		

*Age*				
<15 years	11 (28.95%)	14 (22.58%)	—	0.08
15–30	6 (15.79%)	9 (14.52%)		
30–45	13 (34.22%)	10 (16.12%)		
45–60	4 (10.52%)	11 (17.74%)		
˃60 years old	4 (10.52%)	18 (29.04%)		

*Sex*				
Male	14 (36.84%)	31 (50%)	0.5865 [0.2335; 1.4353]	0.22
Female	24 (63.16%)	31 (50%)		

*Admission*				
Emergency	18 (47.37%)	14 (22.58%)	3.0483 [1.181; 8.0862]	0.01
Programmed	20 (52.63%)	48(77.42%)		

*Venous catheter*				
Yes	16 (42.11%)	24 (38.71%)	1.1499 [0.4646; 2.8274]	0.83
No	22 (57.89%)	38 (61.29%)		

*Associated pathology*				
Yes	5 (13.16%)	12(19.36%)	0.6341 [0.1598; 2.1613]	0.58
No	33 (86.84%)	50 (80.64%)		

*Duration of hospitalization (pre-operative)*				
≤24 h	12 (31.57%)	21 (33.87%)	0.524 [0.1758; 1.4406]	0.25
≥24 h	26 (68.42%)	41 (66.13%)		

*Specialty*				
Child surgery	11 (28.95%)	14 (22.58%)	—	0.726
Gynecology	12 (31.58%)	13 (20.97%)		
Orthopedic surgery	8 (21.06%)	16 (25.80%)		
Visceral	5 (13.15%)	9 (14.52%)		
Urology	2 (5.26%)	6 (9.67%)		
Otolaryngology surgery	—	2 (3.23%)		
Neurosurgery	—	2 (3.23%)		

*Provenance*				
Urban	13 (34.22%)	26 (41.94%)	0.7224 [0.2829; 1.7946]	0.52
Rural	25 (65.78%)	36 (58.06%)		

*SSI*				
Yes	12 (31.58%)	6 (9.68%)	4.2391 [1.3016; 15.3893]	0.007
Women's surgery	2 (16.67%)	1 (16.67)		
Men's surgery	2 (16.67%)	2 (33.33)		
Gynecology	3 (25.00%)	1 (16.67)		
Child surgery	5 (41.66%)	2 (33.33)		
No	26 (68.42%)	56 (90.32%)		
Women's surgery	7 (26.92%)	15 (26.78%)		
Men's surgery	4 (15.39%)	17 (30.36%)		
Gynecology	9 (34.62%)	12 (21.43%)		
Child surgery	6 (23.07%)	12 (21.43%)		

**Table 2:** Demographic and clinical characteristics of patients by nasal carriage status of MRSA and MSSA (*N* = 38).

Demographic and clinical characteristics of patients	Carriers of *Staphylococcus aureus*, *n* = 38		OR confidence interval at 95%	*p* value
	MRSA, *n* = 14	MSSA, *n* = 24		
*Services*				
Surgery for women	2 (14.28%)	7 (29.16%)	—	0.46
Surgery for men	4 (28.58%)	3 (12.50%)		
Gynecology	3 (21.43%)	8 (33.34%)		
Child surgery	5 (35.71%)	6 (25.00%)		

*Age*				
<15 years	5 (35.71%)	6 (25.00%)	—	0.91
15–30	2 (14.29%)	4 (16.67%)		
30–45	4 (28.57%)	9 (37.50%)		
45–60	2 (14.29%)	2 (08.33%)		
>60 years old	1 (07.14%)	3 (12.50%)		

*Sex*				
Male	5 (35.71%)	9 (37.5%)	0.9278 [0.1826; 4.3863]	1
Female	9 (64.29%)	15 (62.5%)		

*Admission*				
Emergency	11 (78.57%)	10 (41.67%)	4.908 [0.9553; 34.6181]	0.04
Programmed	3 (21.43%)	14 (58.33%)		

*Venous catheter*				
Yes	8 (57.14%)	11 (45.83%)	1.5569 [0.3458; 7.3666]	0.73
No	6 (42.86%)	13 (54.17%)		

*Associated pathology*				
Yes	3 (21.43%)	4 (16.67%)	1.3522 [0.1672; 9.6891]	1
No	11 (78.57%)	20 (83.33%)		

*Hospitalization duration (preoperative)*				
≤24 h	4 (25.57%)	11 (45.83%)	0.4821 [0.0853; 2.3066]	0.32
≥24 h	10 (71.42%)	13 (54.17%)		

*Specialty*				
Child surgery	5 (35.71%)	6 (25.00%)	—	0.92
Gynecology	3 (21.43%)	8 (33.34%)		
Orthopedic surgery	3 (21.43%)	6 (25.00%)		
Visceral	2 (14.29%)	3 (12.50%)		
Urology	1 (07.14%)	1 (04.16%)		

*Provenance*				
Urban	3 (21.43%)	10 (41.67%)	0.3913 [0.0556; 2.0501]	0.29
Rural	11 (78.57%)	14 (58.33%)		

*SSI*				
Yes	8 (57.14%)	04 (16.67%)	6.2793 [1.1926; 39.9359]	**0.014**
Women's surgery	1	—		
Surgery for men	2	1		
Gynecology	2	1		
Child surgery	3	2		
No	6 (42.86%)	20 (83.33%)		
Women's surgery	1	7		
Surgery for men	2	2		
Gynecology	1	7		
Child surgery	2	4		

**Table 3:** Antibiotic resistance profile of isolated *Staphylococcus aureus* strains MRSA and MSSA (*N* = 38).

Antibiotics	MRSA (*n* = 14)		MSSA (*n* = 24)	
	Sensitive	Resistant	Sensitive	Resistant
Amikacin (AK)	14 (100%)	—	24 (100%)	—
Amoxicillin + clavulanic acid (AMC)	6 (42.85%)	8 (57.14%)	20 (83.33%)	4 (16.67%)
Amoxicillin (AX)	5 (35.71%))	9 (64.29%)	21 (87.50%)	3 (12.50%)
Chloramphenicol (C)	14 (100%)	—	24 (100%)	—
Ceftazidime (CAZ)	2 (14.28%)	12 (85.72%)	15 (62.50%)	9 (37.50%)
Cefixime (CFM)	3 (21.42%)	11 (78.58%)	16 (66.67%)	8 (33.33%)
Ciprofloxacin (CIP)	14 (100%)	—	24 (100%)	—
Cefalexin (CN)	14 (100%)	—	24 (100%)	—
Ceftriaxone (CRO)	6 (42.85%)	8 (57.15%)	19 (79.17%)	5 (20.83%)
Cefotetan (CT)	12 (85.71%)	2 (14.29%)	24 (100%)	—
Cefotaxime (CTX)	—	14 (100%)	18 (75.00%)	6 (25.00%)
Erythromycin (E)	5 (35.71%)	9 (64.29%)	22 (91.67%)	2 (08.33%)
Fusidic acid (F)	11 (78.57%)	3 (21.43%)	24 (100%)	—
Cefoxitin (FOX)	—	14 (100%)	19 (79.17%)	5 (20.83%)
Imipenem (IPM)	14 (100%)	—	24 (100%)	—
Cephalotine (KF)	2 (14.28%)	12 (85.72%)	14 (58.34%)	10 (41.66%)
Lincomycin (L)	14 (100%)	—	24 (100%)	—
Nalidixic acid (NA)	6 (42.85%)	08 (57.15%)	22 (91.67%)	2 (08.33%)
Ofloxacin (OFX)	14 (100%)	—	24 (100%)	—
Oxacillin (OX)	1 (07.14%)	13 (92.86%)	24 (100%)	—
Piperacillin (PRL)	3 (21.42%)	11 (78.58%)	20 (83.34%)	04 (16.66%)
Rifampicin (RA)	14 (100%)	—	24 (100%)	—
Trimethoprim-sulfametoxazole (SXT)	14 (100%)	—	24 (100%)	—
Tetracycline (TE)	14 (100%)	0—	24 (100%)	—
Tobramycin (TOB)	12 (85.71%)	2 (14.29%)	24 (100%)	—
Ticarcillin (TIC)	—	14(100%)	20 (83.34%)	4 (16.66%)
Teicoplanin (TEC)	4 (28.57%)	12 (85.73%)	21 (87.50%	3 (12.50%)

## Data Availability

All data are available from the corresponding author upon kind request.
